# Ablation lesions in Koch's triangle assessed by three-dimensional myocardial contrast echocardiography

**DOI:** 10.1186/1476-7120-2-27

**Published:** 2004-12-09

**Authors:** Tamas Szili-Torok, Geert-Jan Kimman, Marcoen Scholten, Andrew Thornton, Folkert Ten Cate, Jos Roelandt, Luc Jordaens

**Affiliations:** 1 Department of Cardiology, Thoraxcentre, Erasmus MC, Rotterdam, The Netherlands

## Abstract

**Background:**

Myocardial contrast echocardiography (MCE) allows visualization of radiofrequency (RF) ablation lesions in the left ventricle in an animal model. Aim: To test whether MCE allows visualization of RF and cryo ablation lesions in the human right atrium using three-dimensional echocardiography.

**Methods:**

18 patients underwent catheter ablation of a supraventricular tachycardia and were included in this prospective single-blind study. Twelve patients were ablated inside Koch's triangle and 6, who served as controls, outside this area. Three-dimensional echocardiography of Koch's triangle was performed before and after the ablation procedure in all patients, using respiration and ECG gated pullback of a 9 MHz ICE transducer, with and without continuous intravenous echocontrast infusion (SonoVue, Bracco). Two independent observers analyzed the data off-line.

**Results:**

MCE identified ablation lesions as a low contrast area within the normal atrial myocardial tissue. Craters on the endocardial surface were seen in 10 (83%) patients after ablation. Lesions were identified in 11 out of 12 patients (92%). None of the control patients were recognized as having been ablated. The confidence score of the independent echo reviewer tended to be higher when the number of applications increased.

**Conclusions:**

1. MCE allows direct visualization of ablation lesions in the human atrial myocardium. 2. Both RF and cryo energy lesions can be identified using MCE.

## Introduction

Catheter ablation is a curative treatment for most patients with arrhythmias. In some patients, the results are still suboptimal[1] because of inadequate lesion formation during ablation. Therefore, in these patients direct visualization of ablation lesions may have significant impact on the outcome of the ablation procedures. Direct visualization can also provide additional information for both the development and testing of new dedicated ablation tools. Intracardiac echocardiography (ICE) has been extensively investigated for this purpose [2-6], but the reported results are disappointing[5,7]. Recently, myocardial contrast echocardiography (MCE) has been tested for visualization of ablation lesions in animals in the left ventricle during continuous intracoronary echocontrast infusion[8]. The aim of the present study was to assess the potential use of MCE to demonstrate ablation lesions in human atrial myocardial tissue with continuous venous echocontrast administration.

## Methods

### Patients and study protocol

18 patients were included into this study. The clinical characteristics of the patients are shown in Table 1. All patients underwent EP study and subsequent ablation procedures for supraventricular tachycardia. The Ethics Committee of Erasmus MC, Rotterdam, The Netherlands approved this study. Written, informed consent was obtained. Regardless of the final diagnosis, Koch's triangle was visualized with ICE in all patients at baseline without echocontrast and immediately thereafter, during continuous echocontrast infusion (SonoVue, Bracco). After the ablation procedure, which was either inside or outside Koch's triangle, the ICE procedure was repeated in six patients without echocontrast and in all patients using echocontrast. All ICE procedures were performed using a respiration and ECG gated and triggered pullback technique allowing three dimensional (3D) reconstruction of Koch's triangle. All 2D recordings were analyzed off-line by two independent echocardiographers and they provided confidence scores using a 1–10 grade scale. They were not aware that 6 patients were not ablated in Koch's triangle. This protocol implies that this was a prospective single blind study. 3D reconstruction of the ablation lesions was performed in patients where ablation lesions were seen.

**Table 1:** Clinical characteristics and procedural outcome of the study patients

	Overall group
Number of patients (n)	18
Gender (F/M)	10/8
Age (years ± SD)	49.3 ± 15.7
AVNRT (n)	12
AP (n)	6
Successful ablation (n)	17
Procedure time (min ± SD)	177.1 ± 68.9
Fluoroscopy time (min ± SD)	38.8 ± 27.5
RF/cryo applications (n)	4.2 ± 5.2

n = number, F = female, M = male, AVNRT = atrio-ventricular nodal reentry tachycardia, AP = accessory pathway, min = minutes, RF = radiofrequency, cryo = cryothermy, SD = standard deviation

### Electrophysiology testing and ablation

Standard electrophysiology (EP) and ablation procedures were undertaken using quadripolar electrode catheters in the high right atrium, to record the His bundle electrogram, in the right ventricle and a decapolar diagnostic catheter was inserted into the coronary sinus (CS). The initial portion of the EP procedure was directed at determining the presence of dual AV nodal physiology or accessory pathways, measuring the conduction properties and refractory periods of the fast and slow AV nodal pathways (if present), and determining the mechanism of the paroxysmal SVT. Mapping was performed and after the target site was identified, ablation was applied. Cryothermy and radiofrequency energy were used alternately during the study period.

### Myocardial contrast echocardiography

MCE was performed using SonoVue (Bracco), which is a second generation contrast agent made of microbubbles stabilized by phospholipids and containing sulphur hexafluoride. The mean bubble diameter is 2.5 μm and more than 90% of the bubbles are smaller than 8 μm. The blood level curve shows a distribution half-life of about 1 minute and an elimination half-life of about 6 minutes[9]. In this study we administered SonoVue by continuous intravenous infusion through the cubital vein at a rate of 100 ml/hour. Gain settings were not changed throughout the study.

### Intracardiac echocardiography (ICE)

The ClearView™ system (CardioVascular Imaging Systems Inc, Fremont, CA) was used with an 8F sheath-based ICE imaging catheter that incorporates a 9 MHz beveled single-element transducer rotating at 1800 rpm (model 9900, EP Technologies, Boston Scientific Corp., San Jose, CA, USA).

### ECG- and respiration-gated image acquisition and 3-D image processing

A custom-designed ECG- and respiratory-gated pullback device and a 3D-ultrasound workstation (EchoScan, TomTec GmbH, Munich, Germany) were used to acquire and process the ICE images using a technique described elsewhere[10]. The pullback device is controlled by the 3D workstation and uses a stepping motor to move the catheter stepwise and linearly through the right atrium. The workstation receives video input from the ICE system and an ECG- and respiration-signal (impedance measurement) from the patient. Prior to the acquisition run, the range of RR- and breathing-intervals are measured to define the upper- and lower-limits. The workstation starts acquisition of 2D images after detecting the peak of the R-wave and in the same phase of respiration, at a speed of 25 images/ sec (image interval 40 ms). After acquiring one cardiac cycle, the workstation stores the images, and the catheter is then pulled back by a 0.5-mm axial increment. This process is repeated until the inferior vena cava (IVC) is reached. The acquisition time is much shortened when all cardiac cycles are of the same length, therefore, the right ventricular apex is paced at 100 bpm. In accordance with their timing in the cardiac cycle, all images are formatted in volumetric data sets (256*256*256 pixels/each 8 bits). During post-processing, several algorithms are applied to reduce noise, enhance edges, and reduce spatial artifacts (ROSA filter).

### Statistical analysis

Continuous variables are expressed as mean ± standard deviation. Correlation analysis between the confidence scores and number of ablation lesions were performed using Pearson's test. The level of significance was set at a p value of 0.05.

## Results

### Ablation results (Table 1)

Of the 18 patients undergoing catheter ablation of supraventricular arrhythmias 12 had AVNRT tachycardia. 6 out of these 12 patients were ablated using cryothermy. All but one patient were successfully ablated (one patient with AVNRT). The number of applications in Koch's triangle was 6 ± 4.9, ranging from 1 to 15 applications. The fluoroscopy and procedure times were 45.7 ± 30.8 min and 196 ± 80.2 min, respectively.

### Myocardial contrast echocardiography (Table 2)

**Figure 1:**
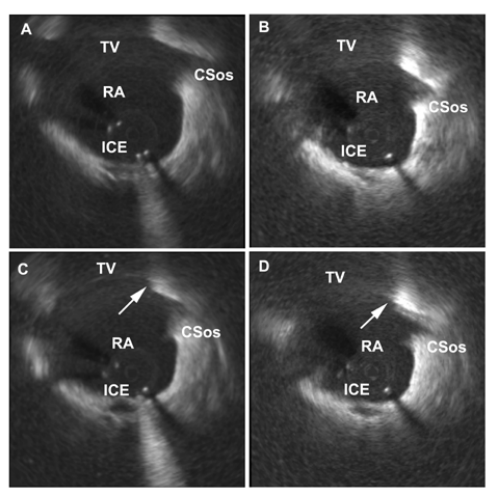
Two-dimensional intracardiac echocardiography images showing part of Koch's triangle between the tricuspid valve and the ostium of the coronary sinus under four different conditions. A: Native 2D horizontal cross-sectional echocardiography image before ablation. B: The same region before ablation with use of echocontrast. C: The same region after radiofrequency energy ablation without echocontrast infusion. A crater as an indirect sign of the ablation lesion (arrow) can be seen on the endocardial surface at the atrial side adjacent to the tricuspid valve. D: The same region after radiofrequency energy ablation and during echocontrast infusion. The ablation lesion (arrow) is visualized as a low contrast area within the atrial myocardial tissue. A crater can be seen on the atrial side adjacent to the tricuspid valve. In both C and D situations (post-ablation) there is significant swelling of the ablated region compared with pre-ablation situations (A and B). ICE = central artifact of the intracardiac echocardiography catheter, TV = tricuspid valve, RA = right atrium, CSos = ostium of the coronary sinus

**Figure 2:**
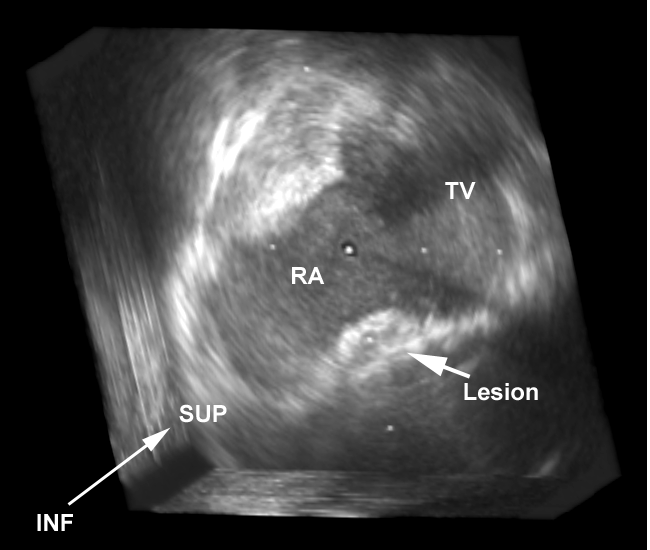
Three-dimensional reconstruction of Koch's triangle: "En face" view of a radiofrequency ablation lesion (arrow). The crater on the right atrial endocardial surface is also well visualized directly to the right. RA = right atrium, TV = tricuspid valve, SUP = superior, INF = inferior

MCE identified ablation lesions as a low contrast area within the normal atrial myocardial tissue (Figure 1). Lesions were identified in 11 out of 12 patients (91%) ablated in Koch's triangle. In only one patient with a single radiofrequency application, was the lesion not recognized. None of the control patients were recognized as having been ablated. The average confidence scores of the independent echo reviewers were 8.5 ± 2.4 and 8.1 ± 2.4, respectively. Both reviewer's confidence scores ranged from 3–10. The confidence score of the independent echo reviewer tended to be higher when the number of applications increased (Reviewer 1: r = 0.697, p = 0.025; Reviewer 2: r = 0.748, p = 0.013). Craters on the endocardial surface were seen in all 12 patients after ablation, by both echo reviewers (Figures 1 and 2).

**Table 2:** Results of myocardial contrast echocardiography in patients ablated in Koch's triangle

		Reviewer 1					Reviewer 2				
Pt. No	Ablation energy	Lesion before ablation		Lesion after ablation		Crater after ablation	Lesion before ablation		Lesion after ablation		Crater after ablation

		NC	C	NC	C		NC	C	NC	C	

1.	RF	-	-	NA	+	+	-	-	NA	+	+
2.	Cryo	-	-	NA	+	+	-	-	NA	+	+
3.	RF	-	-	NA	-	+	-	-	NA	-	+
4.	Cryo	-	-	NA	+	+	-	-	NA	+	+
5.	RF	-	-	NA	+	+	-	-	NA	+	+
6.	Cryo	-	-	NA	+	+	-	-	NA	+	+
7.	RF	-	-	NA	+	+	-	-	NA	+	+
8.	Cryo	-	-	NA	+	+	-	-	NA	+	+
9.	RF	-	-	-	+	+	-	-	-	+	+
10.	Cryo	-	-	-	+	+	-	-	-	+	+
11.	RF	-	-	-	+	+	-	-	-	+	+
12.	Cryo	-	-	-	+	+	-	-	-	+	+

RF = radiofrequency, Cryo = cryothermy, No = number, NC = no contrast (without contrast), C = with contrast, NA = not applicable, Pt = patient

### 3D reconstruction of the lesion

Koch's triangle was successfully reconstructed in 3D in all patients in whom the ablation lesion was previously identified. Lesions could be easily found by 3D echocardiography. An "en face" view of the lesion could be reconstructed in all of these patients (Figure 2).

## Discussion

This study demonstrates that MCE allows visualization of ablation lesions in the human right atrial myocardium during continuous venous echocontrast infusion. This potentially opens a new avenue for objective, and easily accessible evaluation of ablation lesions. Since transmural lesion formation is critical for successful ablation[11], the MCE method may have a significant impact on the outcome of ablation procedures.

### The role of echocardiography in assessment of ablation lesions

Crater formation and tissue changes as increased echo-density have been detected using ICE immediately after RF ablation[12,13]. However, the lesions are not always seen after ablation and indirect signs are being searched for such as changes in local wall thickness[3,4]. The magnitude of the observed changes showed a certain level of correlation with the lesion size. These indirect signs may indicate appropriate lesion formation, but there is an obvious need for direct visualization of the lesions. MCE offers this potential and has allowed visualization of ablation lesions in animals in the left ventricle during continuous intracoronary echocontrast perfusion[8]. The investigators demonstrated high accuracy and reliability in visualizing tissue damage.

Human use does not seem to be practical in this way and neither was safety assessed. In the present study we used continuous peripheral venous echocontrast infusion and we screened lesions in Koch's triangle in humans. We showed in atrial myocardial tissue that the lesions can be visualized and continuous venous infusion provides sufficient differences in echo contrast intensity to directly visualize ablation lesions after focal ablation. In this study we used cryothermy as well as radiofrequency energy for creating lesions in Koch's triangle. We do not have a sufficient number of patients for volumetric comparison of ablation lesions using this method, but it seems that both types of ablation lesion can be reliably visualized using this technique.

### Limitations of the study

This single blind controlled study allowed two independent reviewers to examine the 2D ICE recordings. Although in none of the control patients a lesion in the area of interest was recognized, there was one patient with a single ablation lesion who was not identified by the two independent reviewers. This may suggest that the sensitivity of the technique is still suboptimal. One possible reason is that the concentration of the echocontrast infusion was titrated too low. This is also reflected in the results of the confidence scores. These showed a linear correlation with the number of applications. Furthermore, a correlative study to gross inspection and histopathology would be advantageous. Obviously, this cannot be done in humans.

### The role of 3D reconstruction and future clinical implications

3D reconstruction and a creation of a volumetric data set allow visualization of an "en face" view of the ablation lesion. Without 3D reconstruction determination of the depth and the shape of the ablation lesion was not possible. Therefore the technique could be used to assess whether the ablation lesion was transmural. Furthermore, with 3D reconstruction, the volume of the lesion can be determined. This could potentially be a major asset for clinical electrophysiology since on line 3D echocardiography would allow real-time assessment of ablation lesions. Most importantly, the appropriateness of such lesions could be judged during linear ablations and the continuity of the lines could be checked.

In conclusion, MCE is a safe and promising new method to detect ablation lesion in the human atrial tissue.
